# SARS‐CoV‐2 Spike S2 Refolding: Structural Insights and Inhibition Strategies Against Emerging Variants

**DOI:** 10.1002/mco2.70305

**Published:** 2025-07-15

**Authors:** Chen Xu, Wen Xie, Xinghua Long

**Affiliations:** ^1^ Department of Clinical Laboratory Sir Run Run Shaw Hospital Zhejiang University Hangzhou China; ^2^ Department of Laboratory Medicine Zhongnan Hospital of Wuhan University Wuhan China

1

A recent study in Science reported on the refolding and inhibition of the SARS‐CoV‐2 spike protein (S protein) [1]. It reveals that neutralizing monoclonal antibodies target the conserved refolding machinery of the S2 subunit, blocking critical conformational changes essential for host membrane fusion. The mutating SARS‐CoV‐2 virus enhances immune evasion and drives the resurgence of COVID‐19, necessitating vaccines to prevent infection and counter viral evolution. This report offers new insights for vaccine development.

The study by Grunst et al. provides a compelling exploration of the mechanics of SARS‐CoV‐2, explicitly targeting the refolding of its S protein within host cell membranes. This study employed low‐temperature electron tomography (cryo‐ET) to capture intermediate states during the S2 refolding process and to understand the inhibition of the S2 stem‐helix structure by antibodies (Figure 1). They generated virus‐like particles (VLPs) with retroviral cores derived from human immunodeficiency virus (HIV‐1) and murine leukemia virus (MLV), respectively, which were decorated with either the S protein or angiotensin converting enzyme 2 (ACE2). The advantages of this system lie in its ability to produce high yields of particles carrying either the spike or ACE2. Using the Titan Krios cryo–electron microscope (cryo‐EM), they observed that during the transition from 4°C to 37°C, the spike‐ACE2 complexes largely disappeared, and rod‐like structures emerged, bridging the membrane–membrane interfaces. The subtomogram averaging technique revealed the cross‐linking of ACE2 dimers with the S protein before they transitioned into S2 intermediate states, giving us a better understanding of the SARS‐CoV‐2 entry process. By preventing the refolding of prehairpin intermediates, pan‐β‐coronavirus S2‐targeting antibodies effectively neutralize viral infectivity. The SARS‐CoV‐2 virus continues to circulate within human populations, demonstrating epidemiological persistence even with reduced clinical severity. This work is timely, given the need to understand cross‐reactive immunity against emerging coronavirus variants.

**FIGURE 1:**
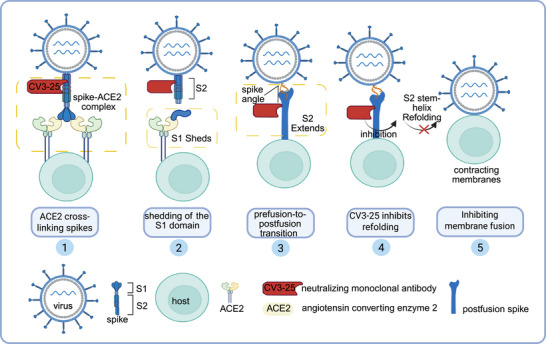
Structure and inhibition of SARS‐CoV‐2 spike S2 refolding by SARS‐CoV‐2 spike (S)‐neutralizing monoclonal antibody, CV3‐25. Image created in BioRender.com.

The S protein, which serves as the principal antigen of the coronavirus, facilitates viral entry by interacting with the host receptor and promoting membrane fusion. Notably, the S proteins of SARS‐CoV‐2 and its phylogenetically related counterpart, SARS‐CoV, exhibit a significant amino acid sequence identity of approximately 77% [2], suggesting the presence of cross‐reactive epitopes. Yuan et al. conducted groundbreaking research by clarifying the structural complex of CR3022, a neutralizing antibody isolated from a patient recovered from SARS‐CoV, with the receptor‐binding domain (RBD) of the SARS‐CoV‐2 S protein. Their findings revealed that CR3022 binds to a conserved epitope shared between SARS‐CoV‐2 and SARS‐CoV, situated distally from the receptor‐binding site. Although the antibody demonstrates cross‐reactivity, its enhanced affinity for SARS‐CoV can be attributed to the presence of a glycan unique to SARS‐CoV's epitope, which is absent in SARS‐CoV‐2. The conservation of this non‐receptor‐binding epitope targeted by CR3022 underscores the potential for developing broadly neutralizing antibodies against SARS‐CoV‐2 and related coronaviruses.

The spike S1 subunit targets most neutralizing antibodies generated in response to SARS‐CoV‐2 infection or spike immunogens. Antibodies that target the RBD of S1 can neutralize SARS‐CoV‐2 by precluding RBD binding to ACE2 [3]. While the spike S2 subunit, particularly its stem‐helix region, exhibits significant conservation across β‐coronaviruses and holds potential as a broad‐spectrum antiviral target, SARS‐CoV‐2 variants consistently acquire mutations in the S1 domain to escape antibody responses elicited by previous infections, vaccinations, or monoclonal treatment therapies [4, 5]. Antibodies and inhibitors that target the stem‐helix show substantial cross‐reactivity against β‐coronaviruses, including SARS‐CoV‐2 variants, SARS‐CoV, and Middle East respiratory syndrome coronavirus (MERS‐CoV).

In this study, the research team utilized cryo‐ET to capture the structure of the SARS‐CoV‐2 S protein binding to the cell receptor ACE2 in the membrane, along with the subsequent conformational changes in the S2 subunit at the base of the protein, uncovering the antiviral mechanism of the stem‐helix region binding neutralizing antibodies. One of the most notable contributions of this study is its structural analysis of the S protein during its interaction with the host membrane. Utilizing a combination of cryo‐EM and molecular dynamics simulations, the authors provide a detailed depiction of the S protein in various conformational states. This groundbreaking work sheds light on the intricate mechanisms underlying S protein refolding and visually demonstrates the process of membrane approximation and fusion between the virus and the host cell.

Emerging variants of concern for SARS‐CoV‐2 demonstrate the capacity to partially evade established immune responses. It is essential to find new antibody therapies and develop new vaccines to combat SARS‐CoV‐2 variants. Li et al. found that the SARS‐CoV‐2 spike (S)‐neutralizing monoclonal antibody, CV3‐25, exerts its antiviral effect by inhibiting membrane fusion through its binding to a highly conserved epitope located within the stem‐helix region of the S2 subunit, which is ubiquitous among β‐coronaviruses. By binding near the HR2 stem‐helix region of prehairpin intermediates, CV3‐25 Fab inhibits the backward movement of HR2 along the HR1 trimer, effectively stabilizing this transitional conformation and preventing membrane approximation. Vaccine immunogens that target conserved epitopes in the RBD and stem‐helix regions could provide broad‐spectrum protective immunity against coronaviruses.

What distinguishes this study is its focus on inhibiting the refolding process. By identifying crucial inhibitors that effectively thwart the S protein's transition into its post‐fusion conformation, the researchers have unveiled a promising avenue for therapeutic intervention and illuminated a pan‐coronavirus antagonistic mechanism. The inhibition strategy outlined in the paper has the potential to pave the way for creating novel antiviral agents capable of blocking the virus' infiltration into host cells. The study provides insights into developing pan‐β‐coronavirus vaccines and S2‐targeted drugs, offering strategies to prevent coronavirus transmission and enhance therapeutic outcomes.

Moreover, the study's methodology is thorough and well‐documented, enabling reproducibility and encouraging future research endeavors by others in the field. Integrating advanced imaging technologies with computational modeling highlights a cross‐disciplinary approach essential for unraveling complex biological phenomena, such as viral pathogenesis. This interdisciplinary synergy ensures a more comprehensive understanding of viral infection mechanisms and lays the groundwork for developing more effective therapeutic strategies.

However, while the study makes significant strides, it also raises questions that warrant further investigation. For instance, the long‐term efficacy of the identified inhibitors and their potential side effects require full exploration. In addition, the variability in membrane composition across different cell types could influence the generalizability of the findings, which is an area that future research should aim to address.

In conclusion, the work by Grunst et al. represents a significant leap forward in virology, providing a deeper understanding of SARS‐CoV‐2 S protein dynamics and a promising foundation for developing new therapeutic strategies. As the fight against COVID‐19 continues, studies like this are crucial in our ongoing efforts to curb the virus' impact and prepare for potential future outbreaks.

## Author Contributions

C.X. drafted the manuscript and drew the figure. W.X. reviewed the manuscript. X.L. drafted and reviewed the manuscript. All the authors read and approved the final manuscript.

## Ethics Statement

The authors have nothing to report.

## Conflicts of Interest

The authors declare no conflicts of interest.

## Data Availability

The authors have nothing to report.

## References

[1] M. W. Grunst , Z. Qin , E. Dodero‐Rojas , et al., “Structure and Inhibition of SARS‐CoV‐2 Spike Refolding in Membranes,” Science 385, no. 6710 (2024): 757–765. 39146425 10.1126/science.adn5658 PMC11449073

[2] P. Zhou , X. L. Yang , X. G. Wang , et al., “A Pneumonia Outbreak Associated With a New Coronavirus of Probable Bat Origin,” Nature 579, no. 7798 (2020): 270–273. 32015507 10.1038/s41586-020-2012-7 PMC7095418

[3] C. O. Barnes , C. A. Jette , M. E. Abernathy , et al., “SARS‐CoV‐2 Neutralizing Antibody Structures Inform Therapeutic Strategies,” Nature 588, no. 7839 (2020): 682–687. 33045718 10.1038/s41586-020-2852-1 PMC8092461

[4] P. Shah , G. A. Canziani , E. P. Carter , and I. Chaiken , “The Case for S2: The Potential Benefits of the S2 Subunit of the SARS‐CoV‐2 Spike Protein as an Immunogen in Fighting the COVID‐19 Pandemic,” Frontiers in Immunology 12 (2021): 637651. 33767706 10.3389/fimmu.2021.637651 PMC7985173

[5] P. Zhou , G. Song , H. Liu , et al., “Broadly Neutralizing Anti‐S2 Antibodies Protect Against all Three Human Betacoronaviruses That Cause Deadly Disease,” Immunity 56, no. 3 (2023): 669–686. 36889306 10.1016/j.immuni.2023.02.005 PMC9933850

